# Incidence and Risk Factors of Postoperative Delirium in Elderly Patients Following Hip Fracture Surgery: A Nationwide Retrospective Cohort Study in Taiwan

**DOI:** 10.1002/gps.70094

**Published:** 2025-05-12

**Authors:** Chien‐An Shih, Deng‐Chi Yang, Wei‐Ming Wang, Yi‐Lin Wu, Yi‐Ching Yang, Han‐Chang Ku

**Affiliations:** ^1^ Department of Orthopedics National Cheng Kung University Hospital College of Medicine National Cheng Kung University Tainan Taiwan; ^2^ Department of Geriatrics and Gerontology National Cheng Kung University Hospital College of Medicine National Cheng Kung University Tainan Taiwan; ^3^ School of Medicine College of Medicine National Cheng Kung University Tainan Taiwan; ^4^ Department of Statistics and Information Science Fu Jen Catholic University New Taipei City Taiwan; ^5^ Department of Nursing National Cheng Kung University Hospital College of Medicine National Cheng Kung University Tainan Taiwan; ^6^ Department of Family Medicine National Cheng Kung University Hospital College of Medicine National Cheng Kung University Tainan Taiwan; ^7^ Department of Nursing Chang Gung University of Science and Technology Chiayi Branch Chiayi Taiwan

**Keywords:** cohort study, delirium, elderly, hip fracture, incidence, surgery

## Abstract

**Background:**

Delirium is an acute cognitive change characterized by behavioral and psychological features, such as visual and auditory hallucinations, sleep disturbances, and emotional confusion. It can lead to extended hospital stays, increased mortality risk, and higher nursing costs. In postoperative hip fracture patients, delirium results in a higher complication rate, poorer functional recovery, increased readmission rates, repeat surgeries, and elevated mortality. Despite these serious consequences, the literature provides limited information on the incidence of postoperative delirium following hip fracture surgeries in Asians. Additionally, there is a lack of long‐term, comprehensive nationwide population‐based studies, highlighting an important area for future research. This study aims to understand the incidence and risk factors of postoperative delirium in hip fracture patients using representative population data.

**Methods:**

We conducted a retrospective cohort study using the Taiwan National Health Insurance Research Database (NHIRD) from 2009 to 2020. The cohort consisted of 118,682 patients aged 65 years or older who were diagnosed with hip fractures. The delirium incidence was observed per 1000 person‐years. The Cox proportional hazards model was used to investigate the incidence of delirium among hip fracture patients.

**Results:**

The incidence of the first episode of delirium after hip surgery in the elderly was 1.87 events per 1000 PYs. Factors associated with delirium included being female (adjusted hazard ratio [aHR]: 0.59; 95% confidence interval [CI]: 0.53–0.64), age ≥ 95 years (aHR: 3.52; 95% CI: 2.74–4.51), comorbid dementia (aHR: 2.63; 95% CI: 2.38–2.92), and ICU stay 2–3 days (aHR: 2.85; 95% CI: 1.28–6.37). The occurrence of delirium was significantly associated with an ICU stay of ≥ 4 days, dementia, as well as 30‐day, 90‐day, and 1‐year mortality (*p* < 0.001).

**Conclusions:**

This study highlights the relatively low incidence of postoperative delirium in elderly hip fracture patients in Taiwan. Key risk factors identified include advanced age, female gender, comorbid dementia, and prolonged ICU stays. These findings underscore the need for targeted prevention and early intervention strategies to improve patient outcomes.

**Summary**

- What is known:
  - Postoperative delirium (POD) is a common yet under‐recognized complication among elderly patients following hip fracture surgery, associated with poor clinical outcomes and elevated mortality.
- What this study adds:
  - Using Taiwan's nationwide population‐based cohort, the incidence of pharmacologically managed POD was 1.87 per 1000 person‐years. Advanced age (≥ 85), female sex, comorbid dementia, and ICU stay ≥ 2 days were significant risk factors for POD.
- Clinical implications:
  - Early identification of high‐risk patients and targeted prevention strategies are essential to reduce the incidence of POD and improve long‐term outcomes, including reducing 1‐year mortality in elderly hip fracture patients.

## 1 Introduction

The global aging population is associated with an increase in the number of hip fractures among individuals aged 65 years and older. In geriatric patients, falling is a common cause of hip fractures, and there has been a rapid upward trend among older adults [1]. Postoperative delirium (POD) is the most common complication after hip fracture [2, 3]. POD is defined as an abrupt and variable impairment in attention and awareness, typically manifesting as daytime sleepiness and night irritability [4]. When patients develop POD, it often becomes the most distressing aspect of the perioperative experience, adding to their personal, medical, and financial burdens [5].

The incidence of POD in elderly patients undergoing hip fracture surgery varies widely, ranging from 10.09% to 51.28%, depending on different countries and study designs [6]. The negative impacts of postoperative delirium in hip fracture patients include higher postoperative complications, poorer functional recovery, increased readmission rates, higher likelihood of repeat surgery, and even elevated mortality rates [7, 8]. However, there is limited literature on the incidence of POD following hip fracture surgeries in Asian populations.

To enable preventive, detective measures against delirium, it is crucial to identify the risk factors for postoperative delirium during hip fracture treatment. Although systematic literature reviews and meta‐analyses indicate that the following risk factors significantly increase the incidence of POD, such as advanced age, age over 80, male gender, lower BMI, hypertension, diabetes, chronic obstructive pulmonary disease, dementia, preoperative functional dependence, visual impairment, smoking, ASA score ≥ 3, regional anesthesia, blood transfusions, hypoalbuminemia, and surgical delay exceeding 48 h [6, 9]. Risk factors for delirium after hip fracture surgeries have been researched in many studies, but the results have been inconsistent [10, 11]. Furthermore, only a few nationwide population‐based studies have been conducted to evaluate the risk factors for POD. These studies have primarily focused on elderly patients undergoing hip fracture surgery under regional anesthesia or restricted to analyzing 20%–25% of patient data in the nationwide database [10, 12].

Our study aims to understand the incidence and risk factors of POD in hip fracture patients in Taiwan using representative population data. We intend to conduct a comprehensive big data analysis to evaluate the risk factors for POD, thereby supporting efforts in preventing POD and managing clinical hip fractures.

## 2 Methods

A retrospective analysis of a population‐based cohort of older adults aged ≥ 65 years who sustained a hip fracture, from January 1, 2009, to December 31, 2020. The present study was exempted from full review by the Internal Review Board of the National Cheng‐Kung University Hospital (No. B‐ER‐111–214).

### 2.1 Data Source

The National Health Insurance Program of the Ministry of Health and Welfare was implemented on March 1, 1995, and has covered more than 99% of the population in Taiwan [13]. The data for this study were obtained from the Health Welfare Data Science Center (HWDC). We used the databases of admissions and outpatient visits, both of which included patient characteristics, including sex and date of birth, the dates of admission, discharge and preventive health service utilization, and three leading diagnoses of outpatients.

The encrypted database encompasses all inpatient and outpatient care claims for patients diagnosed with a hip fracture, identified by the International Classification of Diseases, Ninth Revision, Clinical Modification (ICD‐9‐CM), and Tenth Revision, Clinical Modification (ICD‐10‐CM) codes. A maximum of three leading diagnoses and procedures, details on a participant's date of medical visit, pharmacy refill record, prescription dates, laboratory items, and basic socio‐demographic information, including insured area, and monthly insurance salary, can be obtained. However, the HWDC database was limited by the nature of its retrospective design, and data sources, which do not include clinical characteristics or laboratory parameters, such as medication compliance. The study utilized data from the HWDC Research Database in Taiwan, which is maintained by the National Health Research Institutes (NHRI). The HWDC released de‐identified secondary data to the public for research purposes, and the institutional review board of National Cheng‐Kung University Hospital waived the requirement for informed consent.

### 2.2 The Hip Fracture Cohort

The cohort included patients with a primary or secondary diagnosis of hip fracture, identified by the International Classification of Diseases codes. These include fractures of the femoral neck (ICD‐9‐CM: 820.00–820.09, 820.10–820.19, 820.8–820.9; ICD‐10‐CM: S72.0), intertrochanteric fractures (ICD‐9‐CM: 820.21–820.22, 820.31–820.32; ICD‐10‐CM: S72.1), or subtrochanteric fractures (ICD‐9‐CM: 820.22, 820.32, 820.8; ICD‐10‐CM: S72.2). Additionally, the study includes patients who have undergone hemiarthroplasty, total hip arthroplasty, or appropriate fixation procedures and related surgeries (procedure codes: Hemi/Total arthroplasty (64170B), Hip arthroplasty (64162B), Open reduction internal fixation (64,029B/64028C)) [11, 12]. Exclusions include those with a previous history of hip fracture, those with missing or unknown gender information, those with an observation period of less than 1 year, and those with a prior diagnosis of mental health‐related disorders (ICD‐9‐CM: 291.0–291.9, 292.0–292.9, 303.0–303.9, 304.0–304.9, 305.0–305.9, 295.0–295.9, 298.8–298.9, 297.1–297.3; ICD‐10‐CM: F10–F29). Additionally, patients with multiple traumas or fractures are excluded (ICD‐9‐CM: 906.2, 906.3, V58.89, 828.0–1, 827.0–1, 829.0–1, 839.8–9, 904.9, 922.8, 924.8–9, 959.9; ICD‐10‐CM: S00–S70, S73–S99, T07, T14).

From the Taiwan HWDC, 167,894 individuals underwent hip fracture‐related surgeries between January 1, 2009, and December 31, 2020. Exclusions included 1363 individuals younger than 65 or with an unknown gender, 8782 individuals with mental illness, 39,067 individuals with multiple traumas or fractures, and 128 individuals with uncertain anesthesia codes. Ultimately, the study included 118,554 individuals were included in the analysis.

### 2.3 Research Variables

The primary outcome was the incidence of the first diagnosis of delirium (ICD9_CM: 293.0; ICD10_CM: F05) after hip fracture surgery, consistent with previous claims‐based research definitions [14]. The delirium group comprised all patients with one admission care visit identified through medical claims from the inpatient department. Delirium is deemed present if documented in either the hospitalization or medical order files. In addition to diagnostic coding, we incorporated a pharmacologic confirmation step to enhance case validity. Based on the participant's pharmacy refill record, delirium drug, and prescription dates were retrieved from outpatient and inpatient medical visit claim data. Specifically, patients were considered to have delirium only if they had a corresponding inpatient prescription for at least one antipsychotic medication commonly used for delirium management such as haloperidol (ATC code: N05AD01), risperidone (ATC code: N05AX08), and quetiapine (ATC code: N05AH04) during the same admission [10]. By applying this dual‐criterion approach (diagnosis code plus antipsychotic use), we aimed to reduce potential overestimation of delirium incidence due to misclassification or use of these medications for other psychiatric conditions. No standardized delirium screening tool (e.g., CAM assessments) was applied, as our data source was administrative claims.

Thirty‐day, 90‐day, and 1‐year mortality rates were calculated from the date of admission for the index hip fracture. All inpatient, outpatient, and emergency visits were reviewed to determine 30‐day, 90‐day, and 1‐year mortality through electronic health record review. Data on potential confounders in the association between delirium and subsequent short‐term mortality were also extracted, including age, sex, dementia, ICU stay, reoperation, UTI, pneumonia, wound infection, myocardial infarction, and CVD.

### 2.4 Confounding Variables

Information regarding the potential confounding factors was retrieved from the claim data, including age at hip fracture diagnosis, sex, day of hip fracture surgery, type of surgery, kind of anesthesia, and the event of delirium. Comorbidity included diabetes mellitus (ICD‐9‐CM: 250; ICD10_CM: E10‐E14), hypertension (ICD‐9‐CM: 401–405; ICD10_CM: I10‐I13, I15), hyperlipidemia (ICD9: 272.0–272.4; ICD10: E78.0‐E78.5), coronary artery disease (ICD9: 410–414; ICD10: I20–I22, I24–I25), Parkinson (ICD9: 332.0; ICD10: G20), cerebrovascular disease (CVD) (ICD9: 430–438; ICD10: I60‐I69, G45), dementia (ICD9: 290; ICD10: F00‐F01,F03), Chronic obstructive pulmonary disease (COPD) (ICD9: 490–492, 494–496; ICD10: J44.9), and chronic liver disease (ICD9: 570–571, 573; ICD10: K76.9). The end of the observation date was defined by the occurrence of delirium disorders, withdrawal from the program, or the end of 2020.

### 2.5 Statistical Analysis

Frequencies and descriptive statistics were used to report the baseline characteristics of the variables in our study population. Differences in categorical variables between patients with or without delirium were presented as frequencies with percentages and tested using the chi‐square test. The incidence rate was defined as the number of events per 1000 person‐years (PYs) of observation from 2009 through 2020. Crude hazard ratios, adjusted hazard ratio (aHR), and 95% confidence intervals (95% CI) were calculated using a multivariable Cox proportional hazards regression model, adjusted for sex, age, comorbidities, history of delirium, and ICU admission. Multivariable cox proportional hazards regression model was employed to examine the association of in‐hospital characteristics and mortality, including delirium, age group, gender, ICU ≥ 4 days, dementia, reoperation, infection, urinary tract infection (UTI), pneumonia, wound infection, myocardial infarction, and CVD. Statistical analysis was performed using SAS 9.4 software (SAS Institute, Cary, NC, USA). *p* < 0.05 was considered statistically significant.

## 3 Results

We identified 118,554 patients aged ≥ 65 years who were admitted to the hospital and underwent surgery for hip fracture from 2009 to 2020. The incidence of postoperative delirium was 1.6% (*n* = 1932). Among these patients, 90.8% were aged over 75 years, 51.2% were female, 55.1% underwent open reduction internal fixation, and 47.6% had a Carlson Comorbidity Index (CCI) category of 1–2. The demographic data of the hip fracture cohort with and without delirium are shown in Table 1.

**TABLE 1:** Comparison in demographic characteristics between hip fracture cohort with and without delirium (*N* = 118,554).

	Hip fracture cohort			*p*‐value
	Overall	Delirium (−)	Delirium (+)	
		*n* = 116,622 (98.4%)	*n* = 1932 (1.6%)	
Age, mean (SD), years	81.2 ± 7.4	81.1 ± 7.4	84.6 ± 6.8	**<** **0.001**
Age group				**<** **0.001**
65–74	23,813 (20.1)	23,636 (20.3)	177 (9.2)	
75–84	53,390 (45.0)	52,704 (45.2)	686 (35.5)	
85–94	38,078 (32.1)	37,110 (31.8)	968 (50.1)	
95+	3273 (2.8)	3172 (2.7)	101 (5.2)	
Sex				**<** **0.001**
Male	42,942 (36.2)	41,999 (36.0)	943 (48.8)	
Female	75,612 (63.8)	74,623 (64.0)	989 (51.2)	
Type of fracture				0.002
Femoral neck fracture	62,939 (53.1)	61,925 (53.1)	1014 (52.5)	
Intertrochanteric fracture	54,552 (46.0)	53,666 (46.0)	886 (45.9)	
Subtrochanteric fracture	1063 (0.9)	1031 (0.9)	32 (1.7)	
Type of surgery				0.017
Hemi/Total arthroplasty	49,220 (41.5)	48,358 (41.5)	862 (44.6)	
Hip arthroplasty	302 (0.3)	296 (0.3)	6 (0.3)	
Open reduction internal fixation	69,032 (58.2)	67,968 (58.3)	1064 (55.1)	
Kind of anesthesia				**<** **0.001**
Lumbar (epidural) anesthesia	7017 (5.9)	6945 (6.0)	72 (3.7)	
Lumbar (spinal) anesthesia	70,095 (59.1)	68,959 (59.1)	1136 (58.8)	
General anesthesia	41,442 (35.0)	40,718 (34.9)	724 (37.5)	
Comorbidities				
DM	40,407 (34.1)	39,800 (34.1)	607 (31.4)	0.013
HTN	81,986 (69.2)	80,679 (69.2)	1307 (67.7)	0.149
Hyperlipidemia	21,366 (18.0)	21,079 (18.1)	287 (14.9)	< 0.001
Coronary artery disease	22,414 (18.9)	22,008 (18.9)	406 (21.0)	0.017
Parkinson	6073 (5.1)	5965 (5.1)	108 (5.6)	0.347
Cerebrovascular disease	24,503 (20.7)	24,066 (20.6)	437 (22.6)	0.033
Dementia	13,851 (11.7)	13,307 (11.4)	544 (28.2)	< 0.001
COPD	14,807 (12.5)	14,535 (12.5)	272 (14.1)	0.033
Chronic liver disease	5549 (4.7)	5492 (4.7)	57 (3.0)	< 0.001
CCI Category				**<** **0.001**
0	29,333 (24.7)	28,981 (24.9)	352 (18.2)	
1–2	53,961 (45.5)	53,042 (45.5)	919 (47.6)	
3–6	31,383 (26.5)	30,815 (26.4)	568 (29.4)	
> 6	3877 (3.3)	3784 (3.2)	93 (4.8)	
Ever been to ICU (days)				**<** **0.001**
0	111,018 (93.6)	109,347 (93.8)	1671 (86.5)	
1–3	106 (0.1)	100 (0.1)	6 (0.3)	
4–6	1004 (0.9)	980 (0.8)	24 (1.2)	
≥ 7	6426 (5.4)	6195 (5.3)	231 (12.0)	
Hospital stay (days)	8.9 ± 16.3	8.8 ± 15.2	12.7 ± 47.5	**<** **0.001**

*Note:* Bold values indicate significant difference.

Abbreviations: CCI: Charlson comorbidity index; COPD, chronic obstructive pulmonary disease; DM, diabetes mellitus; HTN, hypertension; ICU: intensive care unit.

The average incidence of the first episode of delirium after the hip fracture surgery was estimated at 1.87 events per 1000 PYs. The average incidence of the first episode of delirium after hip fracture surgery was estimated at 2.39 events per 1000 PYs in males and 1.55 events per 1000 PYs in females. Factors associated with delirium in the hip fracture cohort, based on the multivariate Cox proportional hazards model, included aged 85–94 years ([aHR]: 2.92; 95% CI, 2.48–3.44), aged more than 95 years ([aHR]: 3.50; 95% CI, 2.73–4.48), comorbid dementia ([aHR]: 2.59; 95% CI, 2.34–2.86), ICU stay of ≥ 2–3 days ([aHR]: 2.90; 95% CI, 1.30–6.46), and ICU stay of ≥ 7 days ([aHR]: 2.00; 95% CI, 1.73–2.30), after adjusting for sex, age, comorbidities, history of delirium and ever been to ICU (Table 2).

**TABLE 2:** Cox proportional hazards regression analysis of risk factors for delirium in hip fracture patients.

Variables	Delirium events	Incidence rate	Crude HR	*p*‐value	Adjusted HR^a^	*p*‐value
Total	1932	1.87				
Gender						
Male	943	2.39	—	—	—	—
Female	989	1.55	0.60 (0.55, 0.65)	**<** **0.001**	0.59 (0.53, 0.64)	**<** **0.001**
Age (years)						
65–74	177	0.90	—	—	—	—
75–84	686	1.49	1.73 (1.46, 2.04)	**<** **0.001**	1.60 (1.36, 1.89)	**<** **0.001**
85–94	968	2.81	3.44 (2.91, 4.00)	**<** **0.001**	2.93 (2.49, 3.45)	**<** **0.001**
95+	101	3.34	4.13 (3.23, 5.27)	**<** **0.001**	3.52 (2.74, 4.51)	**<** **0.001**
Kind of anesthesia						
Epidural anesthesia	72	1.14	0.59 (0.46, 0.75)	**<** **0.001**	0.57 (0.45, 0.73)	**<** **0.001**
Spinal anesthesia	1336	2.25	0.93 (0.85, 1.02)	0.132	0.90 (0.82, 0.98)	0.02
General anesthesia	724	1.94	—	—	—	—
Comorbidities						
DM	607	1.67	0.88 (0.80, 0.97)	0.01	1.09 (0.98, 1.21)	0.10
HTN	1307	1.84	0.93 (0.85, 1.03)	0.16	0.96 (0.87, 1.06)	0.46
Hyperlipidemia	287	1.59	0.80 (0.70, 0.90)	**<** **0.001**	0.95 (0.84, 1.09)	0.47
Coronary artery disease	406	1.91	1.14 (1.02, 1.25)	0.02	1.08 (0.97, 1.21)	0.19
Parkinson	108	2.02	1.10 (0.90, 1.33)	0.30	0.92 (0.76, 1.12)	0.40
Cerebrovascular disease	437	1.93	1.12 (1.01, 1.22)	0.04	0.99 (0.88, 1.10)	0.81
Dementia	544	4.23	2.96 (2.68, 3.27)	**<** **0.001**	2.63 (2.38, 2.92)	**<** **0.001**
COPD	272	1.83	1.14 (1.00, 1.30)	0.04	0.89 (0.79, 1.01)	0.07
Chronic liver disease	57	1.05	0.62 (0.47, 0.80)	**<** **0.001**	0.70 (0.54, 0.91)	0.01
Ever been to ICU (days)						
2–3	6	19.93	3.85 (1.73, 8.57)	**<** **0.001**	2.85 (1.28, 6.37)	0.01
4–6	24	4.45	1.61 (1.07, 2.40)	0.02	1.30 (0.87, 1.95)	0.20
≥ 7	231	2.16	2.31 (2.01, 2.65)	**<** **0.001**	1.99 (1.73, 2.29)	**<** **0.001**

*Note:* Bold values indicate significant difference.

Abbreviations: CCI: Charlson comorbidity index; COPD, chronic obstructive pulmonary disease; DM, diabetes mellitus; HTN, hypertension; ICU: intensive care unit.

^a^ Adjusted for sex, age, comorbidities, history of delirium, ever been to ICU (days); incidence rate (1/1000).

**FIGURE 1:**
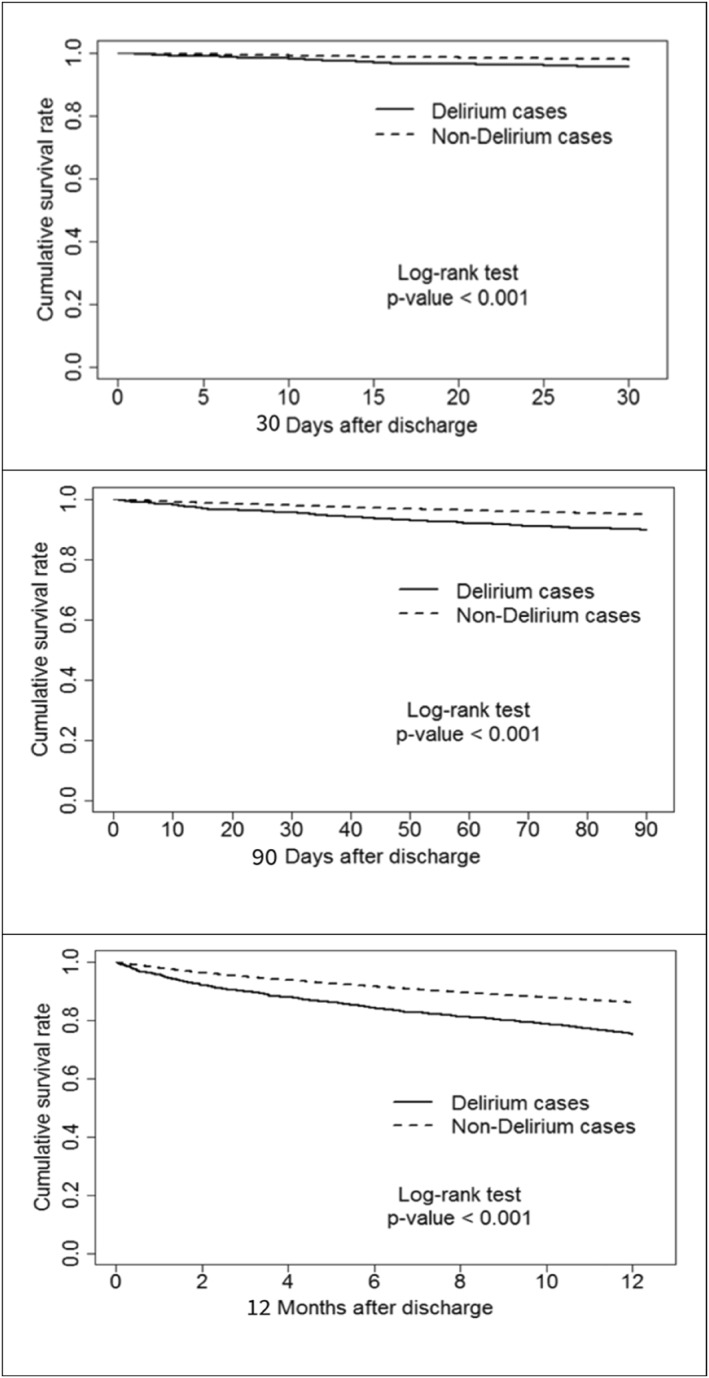
Cumulative survival rate between hip fracture patients with and without delirium.

Clinical outcomes are presented in Table 3. A comprehensive investigation into the impact of delirium on clinical outcomes. Postoperative delirium significantly increased the risk of adverse clinical outcomes within one year, including admission to ICU ≥ 4 days (10.1%), incidence of dementia (35.5%), 30‐day mortality (4.4%), 90‐day mortality (10.1%), and 1‐year mortality (24.8%) (Table 3, Figure 1).

**TABLE 3:** Clinical outcomes within One year after hip fracture surgery.

Variables	Delirium event, No (*n* = 115,037)	Delirium event, yes (*n* = 1860)	*p*‐value
ICU ≥ 4 days	7572 (6.6)	188 (10.1)	**<** **0.001**
Dementia	17,905 (15.6)	660 (35.5)	**<** **0.001**
Reoperation	5376 (4.7)	86 (4.6)	0.92
UTI	265 (0.2)	8 (0.4)	0.09
Pneumonia	83 (0.1)	3 (0.2)	0.16
Wound infection	714 (0.6)	18 (1.0)	0.06
Myocardial infarction	2099 (1.8)	45 (2.4)	0.06
CVD	22,392 (19.5)	372 (20.0)	0.56
Mortality			
30‐day	2324 (2.0)	82 (4.4)	**<** **0.001**
90‐day	5741 (5.0)	187 (10.1)	**<** **0.001**
1 year	16,038 (13.9)	462 (24.8)	**<** **0.001**

*Note:* Bold values indicate significant difference.

Follow‐up for 1 year after discharge showed that the factors associated with 1‐year mortality after POD, based on the multivariate Cox proportional hazards model, included delirium ([aHR]: 1.50; 95% CI, 1.37–1.92), aged over 95 years ([aHR]: 4.14; 95% CI, 3.84–4.48), ICU stay of ≥ 4 days ([aHR]: 4.08; 95% CI, 3.93–4.25), infection ([aHR]: 2.02; 95% CI, 1.09–3.75), and myocardial infarction ([aHR]: 1.39; 95% CI, 1.28–1.51), after confounding sex, age, and comorbidities (Table 4).

**TABLE 4:** Cox proportional hazards regression analysis of risk factors for 1‐year mortality after POD.

Variables	Mortality (%)	Crude HR	*p*‐value	Adjusted HR^a^	*p*‐value
Delirium	462 (24.8)	1.91 (1.74, 2.10)	**<** **0.001**	1.50 (1.37, 1.65)	**<** **0.001**
Gender					
Male	8139 (19.3)	—	—	—	—
Female	8361 (11.2)	0.55 (0.54, 0.57)	**<** **0.001**	0.57 (0.55, 0.59)	**<** **0.001**
Age (years)					
65–74	2037 (8.6)	—	—	—	—
75–84	6506 (12.3)	1.46 (1.39, 1.53)	**<** **0.001**	1.49 (1.41, 1.56)	**<** **0.001**
85–94	6994 (18.8)	2.31 (2.20, 2.92)	**<** **0.001**	2.31 (2.20, 2.43)	**<** **0.001**
95+	963 (30.6)	4.11 (4.48, 4.88)	**<** **0.001**	4.14 (3.84, 4.48)	**<** **0.001**
ICU ≥ 4 days	3361 (43.3)	4.20 (4.04, 4.36)	**<** **0.001**	4.09 (3.93, 4.25)	**<** **0.001**
Dementia	2239 (12.1)	0.80 (0.77, 0.84)	**<** **0.001**	0.70 (0.67, 0.73)	**<** **0.001**
Reoperation	524 (9.6)	0.64 (0.59, 0.70)	**<** **0.001**	0.58 (0.53, 0.63)	**<** **0.001**
UTI	72 (26.4)	1.91 (1.51, 2.40)	**<** **0.001**	1.15 (0.91, 1.15)	0.24
Pneumonia	38 (44.2)	3.57 (2.60, 4.90)	**<** **0.001**	1.14 (0.82, 1.56)	0.44
Wound infection	185 (25.3)	1.90 (1.64, 2.20)	**<** **0.001**	1.68 (1.45, 1.94)	**<** **0.001**
Myocardial infarction	640 (29.9)	2.32 (2.14, 2.51)	**<** **0.001**	1.39 (1.29, 1.51)	**<** **0.001**
CVD	2540 (11.2)	0.72 (0.69, 0.75)	**<** **0.001**	0.66 (0.64, 0.69)	**<** **0.001**

*Note:* Bold values indicate significant difference.

^a^ Adjusted for sex, age, dementia, reoperation, UTI, pneumonia, wound infection, myocardial infarction, CVD.

## 4 Discussion

This study presents a population‐based dataset aimed at determining the incidence of delirium among the hip fracture cohort. Our findings revealed a crude incidence rate of delirium at 1.06%. While the postoperative delirium incidence rate observed in our study is lower compared to that reported in other countries, it remains within the range identified in meta‐analyses. This finding is consistent with two review articles by Albanese et al., and Qi et al. showed an incidence of 4%–53.3% between 1990 and 2021 [5, 6].

In this study, the incidence of postoperative delirium was higher among males than females. Our results were consistent with the findings from three studies, that reported a higher incidence of POD among males compared to females [6, 10, 15, 16]. However, women have a higher fracture incidence [2, 4, 12], and men are more likely to be diagnosed with POD following hip fracture treatment [5, 6, 15, 16].

Our results indicate that the likelihood of developing delirium after hip fracture surgery increases with patient age. The incidence of delirium was significantly higher in older age groups, with age ranges varying considerably across different studies [4, 6, 17]. Our results were consistent with the findings of Ahn et al., which reported that age ≥ 85 years was a predictor of POD [10]. Most previous studies, including ours, found that age ≥ 85 years was a significant predictor of POD [10, 18]. It is further suggested that age ≥ 85 could be the cutoff for delirium prevalence.

Our study found comorbid dementia was associated with a 2.59‐fold increased risk of POD among the hip fracture cohort. This finding was consistent with two cohort studies in the Netherlands [2, 3], which showed that a previous history of dementia was significantly associated with POD. We also found that prolonged ICU stay was associated with a decreased risk of POD. The average incidence of the first episode of delirium after hip fracture surgery was estimated at 19.93 events per 1000 PYs for ICU stays of 2–3 days, 4.45 events per 1000 PYs for ICU stays of 4–6 days, and 2.16 events per 1000 PYs for ICU stays of over 7 days. Urgent surgery before ICU admission is identified as a significant risk factor for the development of delirium [19, 20].

The result was consistent with the findings of Ahn et al., which reported that ICU care was a predictor of POD [10]. The Confusion Assessment Method for the Intensive Care Unit (CAM‐ICU) is the most effective diagnostic tool for delirium assessment in the ICU, enabling rapid identification of delirium at the bedside at least once per nurse practitioner shift [21]. Predicting the development of delirium in critically ill patients upon admission to the ICU and 24 h later is crucial. Early monitoring can promptly identify high‐risk patients, allowing for timely intervention to prevent or reduce the occurrence of delirium [22]. In Taiwan, early monitoring with CAM‐ICU is implemented after ICU admission. The higher incidence of POD for ICU stay of 2–3 days was likely attributable to this early monitoring practice.

Our analysis revealed a statistically significant association between the type of anesthesia and the incidence of POD, with patients receiving general anesthesia exhibiting a higher risk of delirium compared to those undergoing regional anesthesia. This finding aligns with some reports that general anesthesia can increase delirium risk in elderly patients [23]. However, the literature on this issue is mixed. A recent meta‐analysis of randomized trials found no significant difference in POD incidence between general and spinal anesthesia [24, 25]. Thus, the influence of anesthesia technique on delirium remains controversial. Therefore, while our findings may point toward a potential association between anesthesia modality and POD, we advise cautious interpretation. Other unmeasured factors—such as anesthetic depth, specific agents used, or intraoperative hemodynamic fluctuations—may also contribute. Further prospective studies are warranted to clarify the causal role of anesthesia type in POD development.

According to two meta‐analyses of elderly hip fractures, patients with postoperative delirium had more than two‐to threefold the risk of mortality compared to those without delirium [26, 27]. Identifying patients at high risk of POD will enhance both the prevention and treatment strategies, and help improve the hip fracture surgery outcomes. Previous studies have shown that POD is associated with higher complication rates, lengths of hospital stay, cost, and worse prognoses on mortality rates in various types of surgeries [5, 28]. Our study points out that patients who have POD are more likely to mortality, similar to a previous report on hip fractures [2, 16, 29]. Whether POD increases mortality has been debated in the literature. We found that POD may increase 1‐year mortality by 1.50‐fold. Some studies presented an increase in 1‐year mortality and overall mortality [19, 30], other studies controversially demonstrated that delirium was not an independent predictor for mortality [31].

This study has several strengths. Our analysis was based on secondary data from the Taiwan NHIRD; additional factors that could potentially contribute to the observed findings include variances and the duration of exposure within the study samples. It is important to note that the NHIRD encompasses a comprehensive representation of nearly all hip fracture populations in Taiwan, along with their corresponding medical service claim records, including all the diagnostic time, treatment courses, and pharmacy coding. The strength of this study was its use of anonymized data from nearly an entire country's population, which made its results less susceptible to selection bias.

However, our study has some limitations. First, this study used retrospective national claims data, which did not include patient's clinical data. Second, claims data can contain coding errors. Information on diagnosis and disease included in the healthcare utilization database may lack sufficient validity for identifying disease occurrence and prevalence, as the data were analyzed for medical services claims and reimbursements rather than research purposes. Third, the diagnosis of delirium can be easily missed in a clinical setting, or under‐detection due to irregular hospital visits by patients, or because delirium screening is not routinely performed. Additionally, evaluating the nationwide claims data is challenging due to the variable incidence range, potentially leading to underestimations of the true proportion of delirium cases in the study population. Finally, a consistent working definition of delirium is crucial. Further, delirium that does not require pharmacological intervention (such as the hypoactive subtype) may have been omitted in this study; however, the patients receiving haloperidol, risperidone and quetiapine were defined as cases of delirium may overestimate the incidence of delirium because these drugs may have indications other than delirium, so our conclusions should be drawn with caution.

## 5 Conclusions

We retrospectively analyzed data from nearly 120,000 elderly patients who underwent surgery for hip fracture. Our study demonstrated an incidence rate of POD at 1.87 per 1000 PYs for the overall hip fracture study population. Delirium requiring pharmacologic intervention in these patients was associated with multiple risk factors, including female sex, advanced age, comorbid dementia, and ICU stay. Therefore, we recommend emphasizing the importance of early detection of risk factors for POD and implementing adjustments and management strategies that may mitigate the outcomes for patients undergoing hip fracture surgery.

## Author Contributions

Chien‐An Shih designed the study and contributed to revising the article. Deng‐Chi Yang contributed to interpreting the data and revising the article. Wei‐Ming Wang performed the statistical analyses and contributed to revising the article. Yi‐Lin Wu designed the study and contributed to revising the article. Yi‐Ching Yang contributed to revising the article. Han‐Chang Ku designed the study, interpreted the data, wrote the first draft of the article, and contributed to revising the article.

## Ethics Statement

The Institutional Review Board (IRB) of National Cheng‐Kung University Hospital (No. B‐ER‐111‐214), and following the principles of the Declaration of Helsinki.

## Consent

The study utilized data from the Health Welfare Data Science Center (HWDC) Database in Taiwan, which is maintained by the National Health Research Institutes (NHRI). The use of this database was approved by the NHRI and the Bureau of National Health Insurance. Since the data file contained only de‐identified secondary data, the institutional review board of National Cheng‐Kung University Hospital waived the requirement for informed consent.

## Conflicts of Interest

All other authors declare that they have no competing interests relevant to this work.

## Data Availability

Parts of the study findings will be presented in poster format at the 35th International Nursing Research Congress of the Sigma Global Nursing Excellence on July 27, 2024. The data were from the Health Welfare Data Science Center (HWDC) Database in Taiwan, which is maintained by the National Health Research Institutes (NHRI). We were not permitted to share the data.

## References

[1] J. N. Feng , C. G. Zhang , B. H. Li , S. Y. Zhan , S. F. Wang , and C. L. Song , “Global Burden of Hip Fracture: The Global Burden of Disease Study,” Osteoporosis International 35, no. 1 (2024): 41–52, 10.1007/s00198-023-06907-3. 37704919

[2] C. A. Mosk , M. Mus , J. P. Vroemen , et al., “Dementia and Delirium, the Outcomes in Elderly Hip Fracture Patients,” Clinical Interventions in Aging 12 (2017): 421–430, 10.2147/CIA.S115945. 28331300 PMC5354532

[3] E. de Haan , V. A. J. I. M. van Rijckevorsel , P. Bod , G. R. Roukema , and L. de Jong , and Dutch Hip Fracture Registry Collaboration (DHFR) , “Delirium After Surgery for Proximal Femoral Fractures in the Frail Elderly Patient: Risk Factors and Clinical Outcomes,” Clinical Interventions in Aging 18 (2023): 193–203, 10.2147/CIA.S390906. 36818548 PMC9936875

[4] D. Kong , W. Luo , Z. Zhu , S. Sun , and J. Zhu , “Factors Associated With Post‐Operative Delirium in Hip Fracture Patients: What Should We Care,” European Journal of Medical Research 27, no. 1 (2022): 40, 10.1186/s40001-022-00660-9. 35279208 PMC8917680

[5] A. M. Albanese , N. Ramazani , N. Greene , and L. Bruse , “Review of Postoperative Delirium in Geriatric Patients After Hip Fracture Treatment,” Geriatric Orthopaedic Surgery & Rehabilitation 13 (2022): 21514593211058947, 10.1177/21514593211058947. 35282299 PMC8915233

[6] Y. M. Qi , Y. J. Li , J. H. Zou , X. D. Qiu , J. Sun , and Y. F. Rui , “Risk Factors for Postoperative Delirium in Geriatric Patients With Hip Fracture: A Systematic Review and Meta‐Analysis,” Frontiers in Aging Neuroscience 14 (2022): 960364, 10.3389/fnagi.2022.960364. 35992597 PMC9382199

[7] M. S. Haynes , K. D. Alder , C. Toombs , I. C. Amakiri , L. E. Rubin , and J. N. Grauer , “Predictors and Sequelae of Postoperative Delirium in a Geriatric Patient Population With Hip Fracture,” JAAOS: Global Research and Reviews 5, no. 5 (2021): e2000221, 10.5435/JAAOSGlobal-D-20-00221. PMC8133215 33989253

[8] E. J. Jeon and K. Y. Sohng , “Risk Factors and Clinical Outcomes of Delirium After Hip Fracture Surgery in Korean Older Adults: A Retrospective Study,” International Journal of Gerontology 15, no. 1 (2021): 25–29, 10.6890/IJGE.202101_15(1).0005.

[9] J. Wu , Y. Yin , M. Jin , and B. Li , “The Risk Factors for Postoperative Delirium in Adult Patients After Hip Fracture Surgery: A Systematic Review and Meta‐Analysis,” International Journal of Geriatric Psychiatry 36, no. 1 (2021): 3–14, 10.1002/gps.5408. 32833302

[10] E. J. Ahn and S. R. Bang , “Risk Factors Associated With Treatment of Hyperactive Postoperative Delirium in Elderly Patients Following Hip Fracture Surgery Under Regional Anesthesia: A Nationwide Population‐Based Study,” Brazilian Journal of Anesthesiology 72, no. 2 (2022): 213–219, 10.1016/j.bjane.2021.03.020. 33915191 PMC9373072

[11] L. Y. Kuo , P. T. Hsu , W. T. Wu , et al., “The Incidence of Mental Disorder Increases After Hip Fracture in Older People: A Nationwide Cohort Study,” BMC Geriatrics 21, no. 1 (2021): 249, 10.1186/s12877-021-02195-w. 33858356 PMC8051058

[12] J. Poeran , C. Cozowicz , N. Zubizarreta , et al., “Modifiable Factors Associated With Postoperative Delirium After Hip Fracture Repair: An Age‐Stratified Retrospective Cohort Study,” European Journal of Anaesthesiology 37, no. 8 (2020): 649–658, 10.1097/EJA.0000000000001197. 32251149

[13] L. Y. Lin , C. Warren‐Gash , L. Smeeth , and P. C. Chen , “Data Resource Profile: The National Health Insurance Research Database (NHIRD),” Epidemiol Health 40 (2018): e2018062, 10.4178/epih.e2018062. 30727703 PMC6367203

[14] B. Stubbs , G. Perara , A. Koyanagi , et al., “Risk of Hospitalized Falls and Hip Fractures in 22,103 Older Adults Receiving Mental Health Care vs 161,603 Controls: A Large Cohort Study,” Journal of the American Medical Directors Association 21, no. 12 (2020): 1893–1899, 10.1016/j.jamda.2020.03.005. 32321678 PMC7723983

[15] Y. Yang , X. Zhao , T. Dong , Z. Yang , Q. Zhang , and Y. Zhang , “Risk Factors for Postoperative Delirium Following Hip Fracture Repair in Elderly Patients: A Systematic Review and Meta‐Analysis,” Aging‐Clinical & Experimental Research 29, no. 2 (2017): 115–126, 10.1007/s40520-016-0541-6. 26873816

[16] T. Oberai , J. H. F. Oosterhoff , R. Woodman , J. N. Doornberg , G. Kerkhoffs , and R. Jaarsma , “Development of a Postoperative Delirium Risk Scoring Tool Using Data From the Australian and New Zealand Hip Fracture Registry: An Analysis of 6672 Patients 2017‐2018,” Archives of Gerontology and Geriatrics 94 (2021): 104368, 10.1016/j.archger.2021.104368. 33556634

[17] Y. Chen , S. Liang , H. Wu , et al., “Postoperative Delirium in Geriatric Patients With Hip Fractures,” Frontiers in Aging Neuroscience 14 (2022): 1068278, 10.3389/fnagi.2022.1068278. 36620772 PMC9813601

[18] E. M. Kim , G. Li , and M. Kim , “Development of a Risk Score to Predict Postoperative Delirium in Patients With Hip Fracture,” Anesthesia & Analgesia 130, no. 1 (2020): 79–86, 10.1213/ANE.0000000000004386. 31478933 PMC6917900

[19] I. J. Zaal , J. W. Devlin , L. M. Peelen , and A. J. Slooter , “A Systematic Review of Risk Factors for Delirium in the ICU,” Critical Care Medicine 43, no. 1 (2015): 40–47, 10.1097/CCM.0000000000000625. 25251759

[20] S. Renzi , N. Gitti , and S. Piva , “Delirium in the Intensive Care Unit: A Narrative Review,” Journal of Gerontology and Geriatrics 71, no. 1 (2023): 22–29, 10.36150/2499-6564-N600.

[21] J. L. Stollings , K. Kotfis , G. Chanques , B. T. Pun , P. P. Pandharipande , and E. W. Ely , “Delirium in Critical Illness: Clinical Manifestations, Outcomes, and Management,” Intensive Care Medicine 47, no. 10 (2021): 1089–1103, 10.1007/s00134-021-06503-1. 34401939 PMC8366492

[22] M. L. Wang , Y. T. Kuo , L. C. Kuo , et al., “Early Prediction of Delirium Upon Intensive Care Unit Admission: Model Development, Validation, and Deployment,” Journal of Clinical Anesthesia 88 (2023): 111121, 10.1016/j.jclinane.2023.111121. 37058755

[23] S. Zhao , T. Sun , J. Zhang , X. Chen , and X. Wang , “Risk Factors and Prognosis of Postoperative Delirium in Nonagenarians With Hip Fracture,” Scientific Reports 13, no. 1 (2023): 2167, 10.1038/s41598-023-27829-4. 36750657 PMC9905086

[24] G. Fan , M. Zhong , W. Su , et al., “Effect of Different Anesthetic Modalities on Postoperative Delirium in Elderly Hip Fractures: A Meta‐Analysis,” Medicine 103, no. 23 (2024): e38418, 10.1097/MD.0000000000038418. 38847680 PMC11155603

[25] T. Li , J. Li , L. Yuan , et al., “Effect of Regional vs General Anesthesia on Incidence of Postoperative Delirium in Older Patients Undergoing Hip Fracture Surgery: The RAGA Randomized Trial,” JAMA 327, no. 1 (2022): 50–58, 10.1001/jama.2021.22647. 34928310 PMC8689436

[26] Y. Liu , Z. Wang , and W. Xiao , “Risk Factors for Mortality in Elderly Patients With Hip Fractures: A Meta‐Analysis of 18 Studies,” Aging‐Clinical & Experimental Research 30, no. 4 (2018): 323–330, 10.1007/s40520-017-0789-5. 28660596

[27] J. Bai , Y. Liang , P. Zhang , et al., “Association Between Postoperative Delirium and Mortality in Elderly Patients Undergoing Hip Fractures Surgery: A Meta‐Analysis,” Osteoporosis International 31, no. 2 (2020): 317–326, 10.1007/s00198-019-05172-7. 31741024

[28] L. de Jong , V. A. J. I. M. van Rijckevorsel , J. W. Raats , T. M. A. L. Klem , T. M. Kuijper , and G. R. Roukema , “Delirium After Hip Hemiarthroplasty for Proximal Femoral Fractures in Elderly Patients: Risk Factors and Clinical Outcomes,” Clinical Interventions in Aging 14 (2019): 427–435, 10.2147/CIA.S189760. 30880924 PMC6396663

[29] D. H. Lee , C. H. Chang , C. W. Chang , Y. C. Chen , and T. W. Tai , “Postoperative Delirium in Patients Receiving Hip Bipolar Hemiarthroplasty for Displaced Femoral Neck Fractures: The Risk Factors and Further Clinical Outcomes,” Journal of Arthroplasty 38, no. 4 (2023): 737–742, 10.1016/j.arth.2022.10.022. 36273712

[30] E. A. Park and M. Y. Kim , “Postoperative Delirium Is Associated With Negative Outcomes and Long‐Term Mortality in Elderly Koreans: A Retrospective Observational Study,” Medicina 55, no. 10 (2019): 618, 10.3390/medicina55100618. 31547219 PMC6843516

[31] M. G. Kat , J. F. de Jonghe , R. Vreeswijk , et al., “Mortality Associated With Delirium After Hip‐Surgery: A 2‐Year Follow‐Up Study,” Age and Ageing 40, no. 3 (2011): 312–318, 10.1093/ageing/afr014. 21414946

